# Supplementary material for: Tooth for a tooth: Does fighting serve as a deterrent to greater violence in the modern NHL
Source: PLoS One. 2022 Jun 22;17(6):e0269889. doi: 10.1371/journal.pone.0269889 (PMC9216529; doi:10.1371/journal.pone.0269889)
Supplement: S1 Appendix — (DOCX) [file pone.0269889.s001.docx]

**Appendix**

| Type of penalty | Minor | Major | Misconduct |
| --- | --- | --- | --- |
| Violent | Boarding  Charging  Cross-checking  Check to head  Elbowing  Head-butting  High-sticking  Kneeing  Roughing  Spearing | Boarding  Charging  Cross-checking  Check from behind Check to head  Elbowing  Head-butting  High-sticking  Interference  Kneeing  Roughing  Slashing  Spearing | Game misconduct  Head-butting  Match penalty |
| Tactical/Other | Bench  Broken stick  Closing hand on puck  Delay of game  Diving/Embellishment  Goalie interference  Holding  Holding the stick  Hooking  Interference  Penalty shot  Throwing stick  Tripping | N/A | 10-minute misconduct |
